# Supplementary material for: Reporting health services research to a broader public: An exploration of inconsistencies and reporting inadequacies in societal publications
Source: PLoS One. 2021 Apr 7;16(4):e0248753. doi: 10.1371/journal.pone.0248753 (PMC8026015; doi:10.1371/journal.pone.0248753)
Supplement: S2 Appendix — (DOCX) [file pone.0248753.s002.docx]

**S2 Appendix**

**Coding scheme**

| **Date** |
| --- |
| After publication |
| Before publication |
| **Messages** |
| Inconsistency |
| consistency |
| Literal repetition of the title |
| **Inconsistency** |
| Objectives not included in sci pub |
| Effect size exaggeration |
| Generalisation |
| Generalisation / time periods |
| Generalisation / setting or institutions |
| Generalisation / populations |
| Generalisation / geographical location |
| Causality |
| Implications |
| Recommendations |
| Entire recommendation is not included |
| Possible limitations of the recommendation is not mentioned |
| part of the recommendation is not included |
| Bias is left out |
| Strengths of study are not described in sci.pub |
| Discussion in SP does not include mentioned limitations |
| Rhetorical version of the conclusion |
| Conclusion is different in the scientific publication |
| Interpretation is not included in the scientific publication |
| Misinterpretation of outcome |
| conclusion is not included in scientific publication |
| Part of conclusion is not included in scientific publication |
| The conclusion is not based on the results |
| Stronger statement than the results and conclusion may allow |
| Message has no basis in scientific publication |
| Introduction evidence is stated as conclusion |
| Message contradicts findings from the scientific study |
| Results not included in this combination |
| result not included in scientific publication |
| Result is different from the scientific publication |
| Non significant result is presented as a significant result |
| **No Reporting inadequacies** |
| **Replicated reporting inadequacies** |
| Causality |
| Generalisation to geographic locations |
| Implications |
| Abstract conclusions |
| Sampling generalisation |
| Results in the abstract |
| Title |
| Conclusion as based on results |
| Recommendation |
| **Connection to the author** |
| Institute / funder is author |
| Author is primary author |
| No direct involvement authors |
